# Expression of interleukins in adult female acne: evaluating the effects of azelaic acid

**DOI:** 10.1093/skinhd/vzag023

**Published:** 2026-05-04

**Authors:** Diogo Pazzini Bomfim, Cristiane Damas Gil, Marco Rocha, Angel Alvarez Santos, Mílvia Maria Simões e Silva Enokihara, Ediléia Bagatin

**Affiliations:** Department of Medicine, Federal University of São Paulo (Unifesp), Translational Medicine, São Paulo, Brazil; Department of Histology, Federal University of São Paulo (Unifesp), São Paulo, Brazil; Department of Dermatology, Federal University of São Paulo (Unifesp), São Paulo, Brazil; Department of Pathology, Federal University of São Paulo (Unifesp), São Paulo, Brazil; Department of Pathology, Federal University of São Paulo (Unifesp), São Paulo, Brazil; Department of Dermatology, Federal University of São Paulo (Unifesp), São Paulo, Brazil

## Abstract

**Background:**

Adult female acne (AFA) is a multifactorial skin condition that affects women aged > 25 years, with inflammation playing a central role in its aetiopathogenesis. However, most research has primarily focused on severe acne vulgaris, while the immunological mechanisms underlying AFA remain poorly understood.

**Objectives:**

To analyse interleukin (IL)-6 and IL-8 expression in inflammatory lesions of AFA, before and after treatment with topical azelaic acid (AZA) 15% gel, and compare the findings with healthy skin; and to evaluate the efficacy of AZA based on clinical response and histopathological findings.

**Methods:**

Twenty women with mild-to-moderate AFA used AZA 15% gel twice daily for 6 months, with monthly follow-ups. Skin biopsies from lesional and healthy facial areas were collected at baseline and at treatment completion to assess IL-6 and IL-8 by immunohistochemistry and to score inflammation. Clinical evaluation, including Visia^®^ imaging, Investigator Global Assessment and the Erythema Assessment Scale, was performed before and after treatment.

**Results:**

There was significant overexpression of IL-6 and IL-8 in acne lesions compared with healthy skin, *P* < 0.05. Following treatment, a significant reduction in IL-6 (*P* < 0.0001) and IL-8 (*P* < 0.05) expression was observed. Skin with acne lesions exhibited intense inflammatory infiltrate and a significant reduction post-treatment. Treatment with AZA resulted in highly significant clinical improvement of acne severity and erythema (*P* < 0.0001).

**Conclusions:**

IL-6 and IL-8 expressions are increased in AFA, contributing to its inflammatory process. Therapies that reduce these proinflammatory cytokines, such as AZA, are beneficial. Our findings are consistent with a potential anti-inflammatory effect of AZA, given the observed decrease in IL-6 and IL-8.

## Teaser

Adult female acne (AFA) is a multifactorial skin condition affecting women over the age of 25, with inflammation playing a central role in its etiopathogenesis. However, most research has primarily focused on severe acne vulgaris, while the immunological mechanisms underlying AFA remain poorly understood. Our study showed that the expression of interleukin (IL)-6 and IL-8 is increased in AFA, contributing to its inflammatory process. Therapies that reduce these proinflammatory cytokines, such as azelaic acid (AZA), are beneficial. This study highlights AZA’s anti-inflammatory effects, demonstrated by its ability to downregulate IL-6 and IL-8.

What is already known about this topic?

- Inflammation is central to acne development; cytokines involved in the differentiation and maintenance of T helper 17 cells [interleukins (IL)-1β, IL-6 and IL-23, and tumour necrosis factor] trigger acne-related pathogenic processes and intensify inflammation.
- IL-8 acts as a key proinflammatory cytokine and chemoattractant.
- Research on interleukins in adult female acne (AFA) is limited.

What does this study add?

- IL-6 and IL-8 levels are elevated in acne lesions among adult women; after 6 months of azelaic acid (AZA) treatment, IL-6 and IL-8 levels decreased, indicating reduced inflammation.
- The treatment demonstrated therapeutic efficacy, confirmed by Investigator Global Assessment.
- The study furthers understanding of inflammatory mechanisms in AFA; clinical applications include identifying potential new targets, in this case IL-6 and IL-8, to improve treatment.

Adult female acne (AFA) affects up to 20% of women over the age of 25 years^1^ and has increasingly been recognized as a condition distinct from adolescent acne. Rather than a simple continuation of acne vulgaris (AV), AFA represents a unique clinical entity, shaped by its own biologic triggers and patterns of inflammation, and has a meaningful psychosocial burden.^2^ Many women describe its onset as unexpected and discouraging: a recurrence of acne they believed they had outgrown, or a new condition emerging for the first time in adulthood.

Biologically, AFA arises from a complex interplay of hormonal-, immunological-, microbial- and skin barrier-related mechanisms. Hormonal fluctuations, cosmetic use, psychological stress and genetic predisposition further modulate sebaceous gland activity and amplify inflammatory responses, contributing to the marked clinical heterogeneity observed in adult women.^3^

Unlike AV, which frequently involves prominent seborrhoea and comedone formation, AFA is characterized by persistent, low-grade inflammation.^4,5^ This inflammatory state is driven by enhanced sensitivity of sebaceous glands to normal androgen levels and by amplified inflammatory signalling within the pilo­sebaceous unit.^5^ These factors help sustain cytokine release and chronic lesion development, distinguishing AFA from the more fluctuating patterns of AV.

The immunological mechanisms underlying acne, particularly AFA, remain incompletely understood. Most available data are derived from studies of AV, which show involvement of both innate and adaptive immune pathways. These studies demonstrate that infiltrating immune cells are activated by *Cutibacterium acnes* and *Staphylococcus epidermidis* within hair follicles, through mechanisms involving virulence-associated genetic elements, altered transcriptional activity, changes in lipid composition and neuropeptide signalling.^6^ Among the cytokines implicated, interleukin (IL)-6 and IL-8 are of particular interest: they are secreted by macrophages, keratinocytes, peripheral blood mononuclear cells and sebocytes in response to *C. acnes* and substance P, and elevated levels have been consistently observed in acne lesions.^6^ However, despite this evidence, the specific roles of IL-6 and IL-8 in the pathophysiology of AFA remain poorly defined, representing an important gap in current understanding and an area requiring further investigation.

Therapeutic options for AFA include topical agents, isotretinoin, antibiotics and hormonal therapies such as oestrogens, progestins and spironolactone. Azelaic acid (AZA) is a well-established, nonteratogenic treatment for mild-to-moderate AFA, with anti­microbial, anti-inflammatory, depigmenting and mild comedolytic properties.^7^ It is also beneficial for postacne erythema (PAE) and acne-induced macular hyperpigmentation (AMH), and is recommended as a nonantibiotic option to help limit antimicrobial resistance.^7,8^ Initially identified as a tyrosinase inhibitor, AZA also exhibits bacteriostatic activity against *C. acnes* and may inhibit 5-alpha-reductase *in vitro*.^9,10^

This study aimed to better understand the pathophysiology of AFA, focusing on IL expression in inflammatory skin lesions, and to observe any modifications after treatment with AZA.

## Materials and methods

This was a randomized, comparative diagnostic and therapeutic intervention study in translational medicine. The trial was registered at ensaiosclinicos.gov.br with identifier: 4.679.887.

### Study population

This prospective study included 20 women aged 25–44 years with mild (≤10 lesions) to moderate (11–20 lesions) papulopustular facial acne, from May 2021 to August 2022 at the Dermatology Outpatient Department of the Federal University of São Paulo (UNIFESP), São Paulo, Brazil. Patients who had used any topical or systemic acne treatment in the past 6 months, or were pregnant or lactating, were not included in the study. Additionally, individuals receiving steroid derivatives, lithium, anticonvulsants, isoniazid, oral contraceptives, androgens, danazol, iodides, bromides, disulfiram, cyclosporine, azathioprine, thioureas or vitamins B2, B6 or B12, or with a history of hypersensitivity to one of the components of azelaic acid, were also ineligible.

The sample size was determined by the study’s statistical feasibility and operational constraints. Given the complexity of performing skin biopsies in adult female acne and the fact that a single researcher conducted all laboratory procedures, the chosen sample size was considered adequate for the study’s objectives.

### Treatment

Participants applied AZA gel 15% (Azelan^®^, LEO Pharma, São Paulo, Brazil) twice daily for 6 months, using a pea-sized amount to cover the entire face, extending to the submandibular area. They also used standardized gentle skin hygiene products and a moisturizing sun protection factor 30 sunscreen for acne-prone skin. Monthly visits monitored adherence, adverse events and exclusion criteria, with additional 3- and 6-month follow-ups to assess adverse events and relapses.

### Biopsy and pathological evaluation

At study baseline, a 3-mm punch biopsy was performed on an inflammatory lesion (erythematous papule) in the submandibular region under local anaesthaesia. Additionally, another biopsy sample of normal facial skin (perilesional area) was taken from an adjacent area to serve as a control. At the treatment endpoint, a third skin biopsy was obtained from a resolving (healed) acne lesion.

Immunohistochemistry was performed according to standard methods using EnVision^®^ FLEX reagents (DAKO, Carpinteria, CA, USA) and manufacturer-recommended conditions. IL-6 (1:50) and IL-8 (1:100) polyclonal antibodies in rabbits (Novus Biologicals, Centennial, CO, USA) were applied after antigen retrieval in high-pH solution, and visualization was achieved with DAB chromogen.

The stained slides were observed under an Axioscope.A1 optical microscope (ZEISS, Oberkochen, Germany), using Image J (National Institutes of Health, Bethesda, MD, USA) and densitometry. The intensity of the marking was read by the colour histogram in the ‘red’ channel, ranging from 0 (black) to 255 (white) arbitrary units. Lower values indicate more intense marking or greater expression of the marker. Approximately 350 images from the sample were analysed using photomicrographs.

The inflammatory score was performed using photomicrographs obtained with a ×20 objective lens, as follows: absent (0); mild (1), ≤25% of the field with inflammatory infiltrate in tissue; moderate (2), 26–50% of the affected field; or intense (3), ≥50% of the affected area.^11^

All histological analyses were conducted in ImageJ, and clinical evaluations were performed under blind conditions.

### Clinical evaluation

Acne severity, evaluated using Investigator Global Assessment (IGA), was ascertained at baseline and after 6 months of treatment. Acne was graded (grade 0–4) using the simple US Food and Drug Administration 5-category global system of acne classification based on predominant lesions.^12^ Erythema was evaluated using the Erythema Assessment Scale, part of the IGA and Clinician Erythema Assessment (CEA) scales, both scored on a 5-point rating scale (0–4) for severity of facial erythema.^13^

To improve the reliability of the clinical assessment, the examination was further supported by imaging. Photographs were taken on the day of inclusion, and again after 6 months of treatment, using Visia® (Medsystems®, São Paulo, Brazil). This system uses a rotating software camera module, which simplifies image capture, improving patient comfort and diagnostic accuracy. Visia® is a widely used, effective, noninvasive measuring instrument manufactured by Canfield Scientific (Parsippany, NJ, USA). The system captures high-resolution images and simultaneously analyses facial skin parameters, including melanin and haemoglobin, offering insights into sun damage and microvascular dilation.^14^ Noninvasive tools like Visia^®^ are valuable for detecting erythema before it becomes clinically visible and for providing quantitative measures to support diagnosis and monitor treatment response.^15^

Participants washed their faces with water for 30 s before imaging. The room temperature was maintained at 21 ± 1 °C, and frontal, left and right facial images were captured.

### Statistical analysis

A descriptive analysis was performed for baseline data. Categorical variables were presented as absolute and relative frequencies, while quantitative variables were expressed as mean (SD) when normally distributed, or as median with quartiles and minimum–maximum values when normality assumptions were not met.

Inflammatory scores were compared using one-way anova, with pairwise comparisons evaluated using the Bonferroni post hoc correction. Comparisons of clinical assessments before and after treatment were performed with the Kruskal–Wallis test, and pairwise differences were examined using Dunn’s post hoc test. Adverse events were described as absolute and relative frequencies.

A two-sided significance level of 5% (α = 0.05) was adopted for all analyses.

## Results

Twenty women aged 25–44 years with mild-to-moderate AFA were included in the study. The characteristics of the study population are presented in Table 1. Three patients withdrew from the study due to personal reasons, including changing jobs or cities, work commitments or being unable to travel to the study site. None of these withdrawals was related to the treatment.

**Table 1:** Baseline characteristics of study population (*n* = 20)

Age (years), mean (SD)	33.4 (5.77)
Persistent acne (%)	17 (85)
Late-onset acne (%)	3 (15)
Tobacco use (%)	1 (5)
Alcohol use (%)	12 (60)
Family history of acne (%)	14 (70)
Family history of metabolic syndrome (%)	19 (95)
Obesity (%)	14 (70)
Never consulted dermatologist (%)	6 (30)
Treated for short periods only (%)	15 (75)
Acne severity: mild (%)	12 (60)
Acne severity: moderate (%)	8 (40)

**Figure 1:**
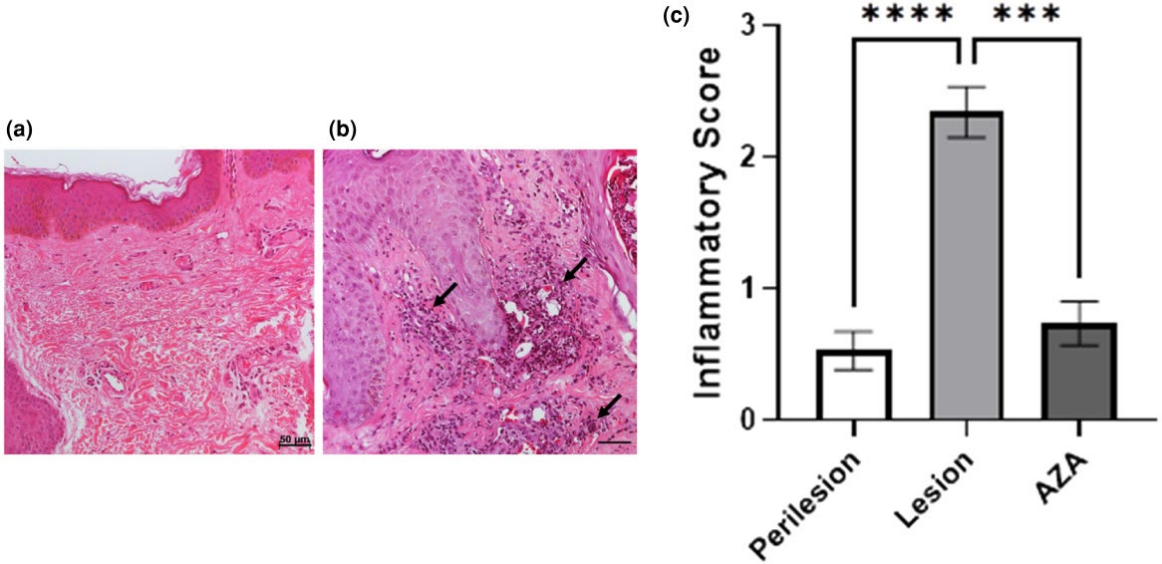
Histological analysis. (a) Perilesional area, showing normal-appearing epidermis and dermis. (b) Acne lesion characterized by intense inflammatory infiltrate in dermis (arrows). Staining: haematoxylin and eosin. Scale bar: 50 µm. (c) Inflammatory score. Data expressed as mean (SD) of inflammatory score. AZA, azelaic acid. ***P < 0.001, ****P < 0.0001, (anova, Bonferroni post hoc test).

Fifty-four skin biopsies were evaluated. In the analysis of haematoxylin and eosin slides, skin with acne lesions showed an intense dermal inflammatory infiltrate compared with healthy skin, which was significantly reduced post-treatment. Mean inflammatory score was 0.5294 in perilesional areas, 2.344 in lesional areas and 0.7353 in lesional areas treated with AZA (*P* < 0.001) (Figure 1).

**Figure 2:**
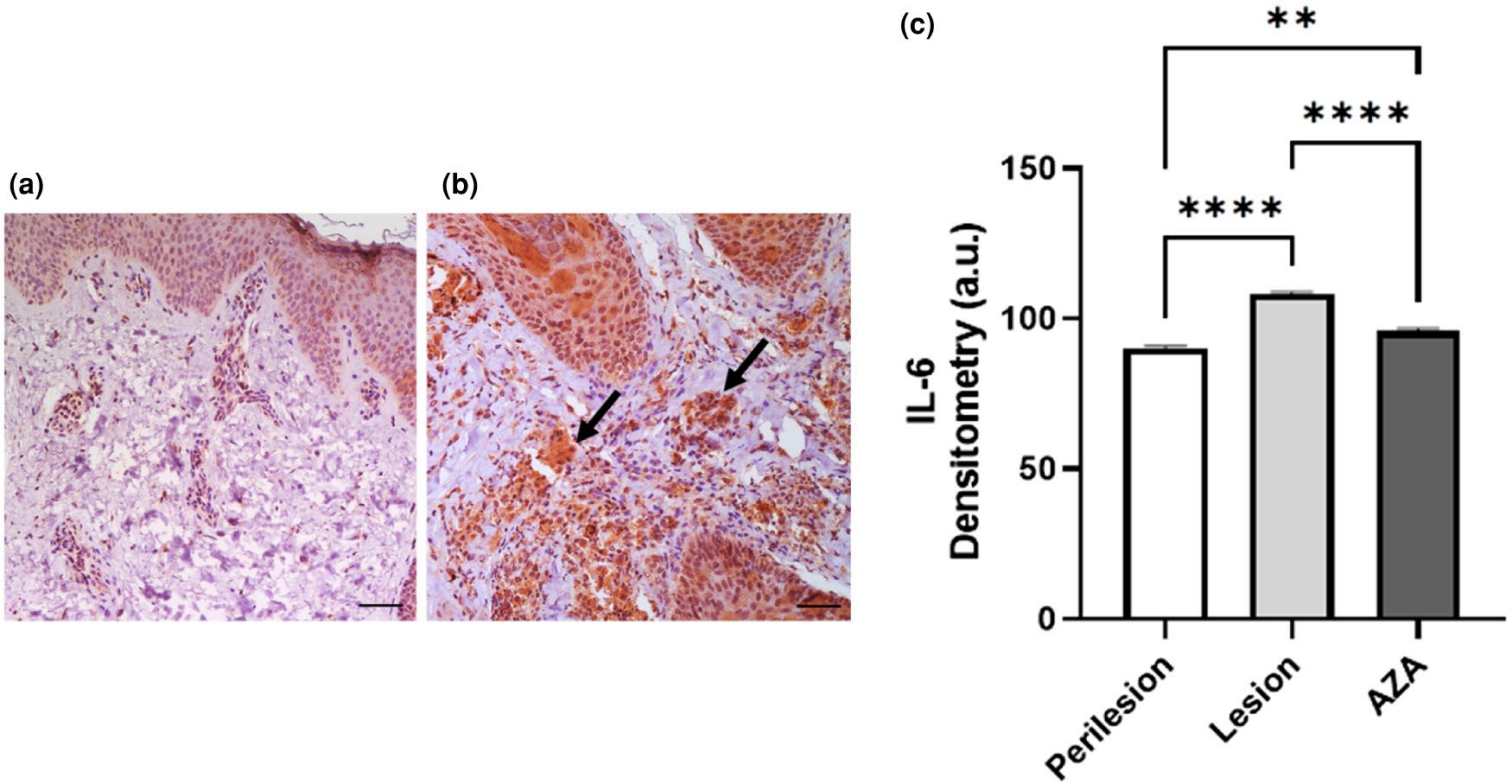
Interleukin (IL)-6 expression in skin. (a) Perilesional area, showing healthy-appearing epidermis and dermis. (b) Acne lesion. Intense immunoreactivity for IL-6 in inflammatory infiltrates (arrows) of lesional skin (b) relative to healthy skin (a). Counterstain: haematoxylin. Scale bar: 50 µm. (c) Densitometry of IL-6 expression in skin. Data shown as mean (SD) of cytokine expression, in arbitrary units (a.u.). AZA, azelaic acid. **P < 0.01, ****P < 0.0001.

There was marked overexpression of IL-6 in acne lesions compared with healthy skin, with significant reduction after treatment (*P* < 0.0001) (Figure 2). There was no significant difference in IL-6 expression between participants with mild acne and those with moderate acne (data not shown).

**Figure 3:**
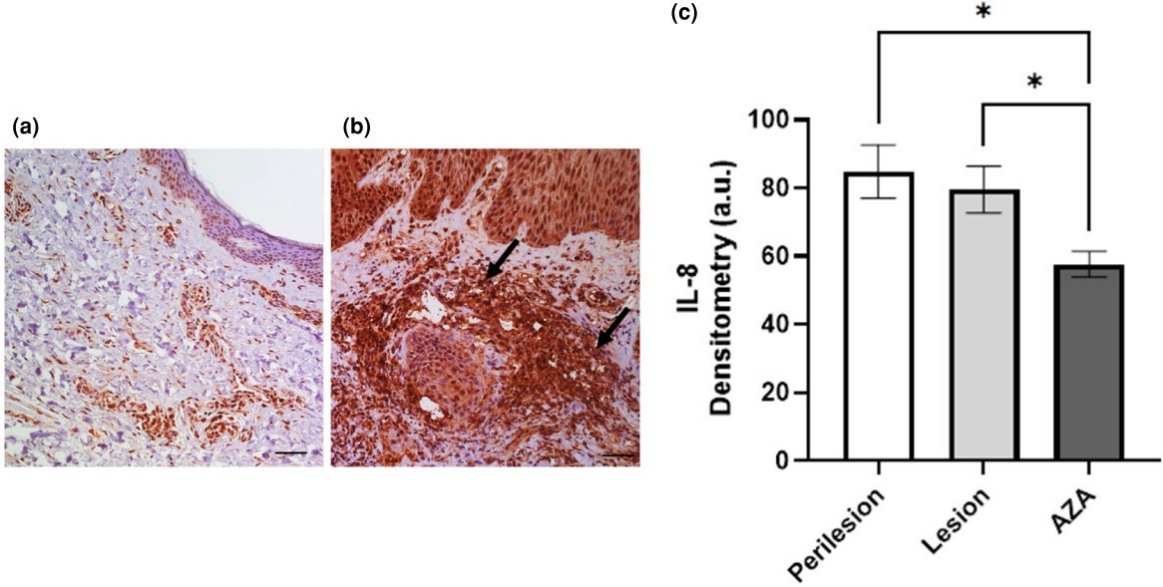
Interleukin (IL)-8 expression in skin. (a) Perilesional area. (b) Acne lesion. Intense immunoreactivity for IL-8 in inflammatory infiltrates (arrows) of lesional skin (b) relative to healthy skin (a). Counterstain: haematoxylin. Scale bar: 50 µm. (c) Densitometry of IL-8 expression in skin. Data shown as mean (SD) of cytokine expression, in arbitrary units (a.u.). AZA, azelaic acid. *P < 0.05.

Similarly, IL-8 was expressed at higher levels in acne lesions than in healthy skin, and this expression was significantly reduced following treatment (*P* < 0.05) (Figure 3). There was no significant difference in IL-8 expression between participants with mild and moderate acne (data not shown).

Clinical treatment with AZA resulted in significant clinical improvement after 6 months, according to IGA score (Table 2), IGA for erythema and CEA scores (Table 3) (*P* < 0.001).

**Table 2:** Distribution of Investigator Global Assessment (IGA) acne severity scores at baseline and at the end of treatment

IGA score	Baseline (%)	Treatment endpoint (%)
0	0	64.7
1	17.6	35.3
2	47.1	0
3	35.3	0
4	0	0

IGA scores: 0 = clear; 1 = almost clear; 2 = mild; 3 = moderate; 4 = severe. Percentages are based on 17 participants at each timepoint, as three participants were excluded.

**Table 3:** Erythema assessment according to Investigator Global Assessment (IGA) and Clinician Erythema Assessment (CEA) scores at baseline and at the treatment endpoint

IGA score			
Baseline	Endpoint: clear	Endpoint: mild	Total
Almost clear	7 (100.00)	0 (0.00)	7 (100)
Mild	3 (75.00)	1 (25.00)	4 (100)
Moderate	1 (25.00)	3 (75.00)	4 (100)
Moderate–severe	1 (100.00)	0 (0.00)	1 (100)
Total	13 (76.00)	4 (24.00)	17 (100)
**CEA score**			
**Baseline**	**Endpoint: clear**	**Endpoint: almost clear**	**Total**
Clear	11 (100.00)	0 (0.00)	11 (100)
Almost clear	2 (100.00)	0 (0.00)	2 (100)
Moderate	2 (66.67)	1 (33.33)	3 (100)
Total	16 (94.11)	1 (5.89)	17 (100)

Values represent the number of participants (%) within each baseline category who achieved the corresponding endpoint severity rating. Percentages are based on 17 participants at each timepoint, as three participants were excluded.

**Figure 4:**
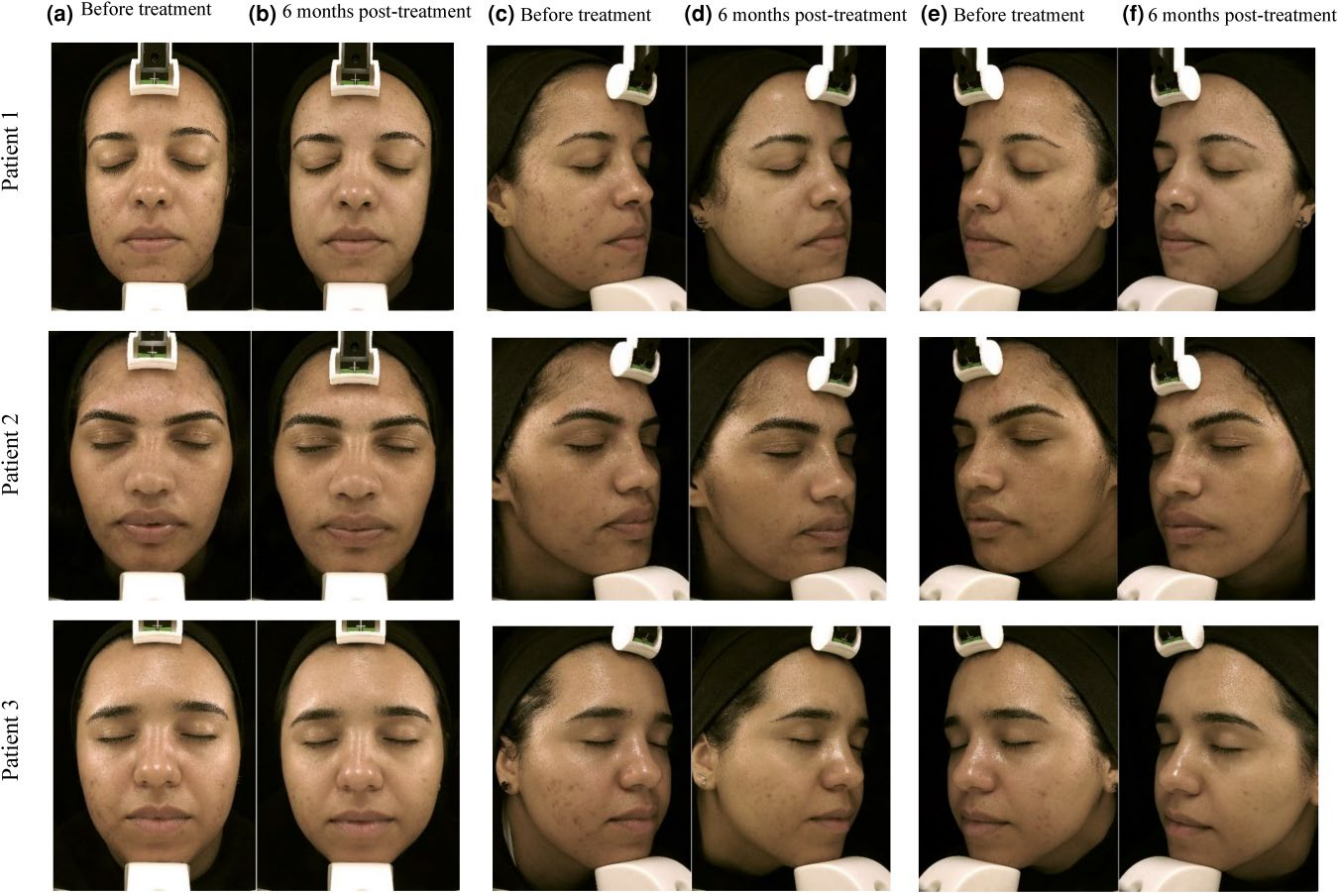
Three patients from the study: clinical improvement to (a, b) front, (c, d) right-hand and (e, f) left-hand side of face, as demonstrated by Visia^®^ at baseline and 6 months after use of azelaic acid 15% gel twice daily.

**Figure 5:**
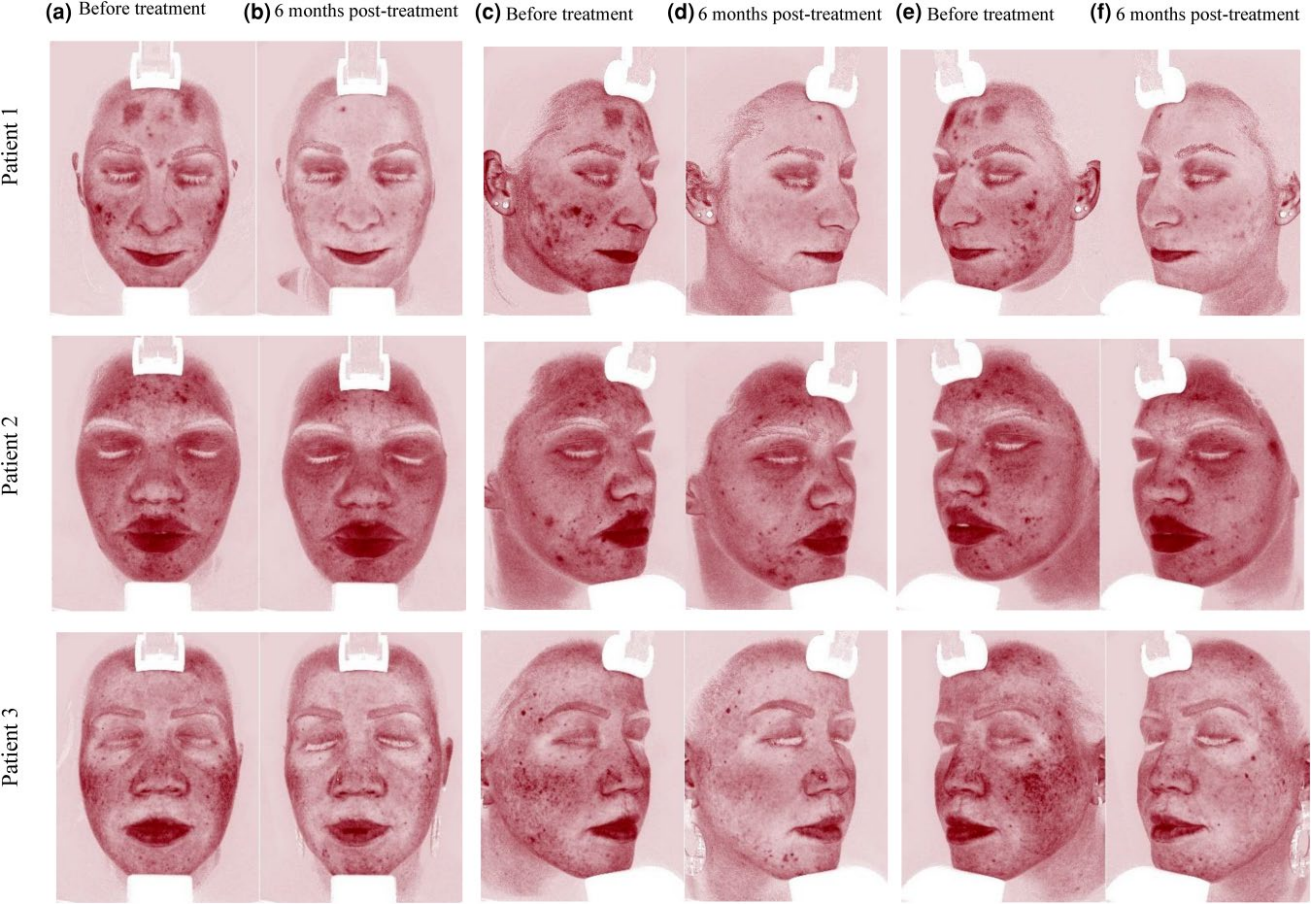
Three patients from the study: erythema improvement to (a, b) front, (c, d) right-hand and (e, f) left-hand side of face, as demonstrated by Visia^®^ at baseline and 6 months after use of azelaic acid 15% gel twice daily.

Figure 4 illustrates three patients before and after treatment, showing a reduction in acne lesions and improved AMH at the endpoint. Figure 5 shows three patients before and after treatment, with improvement in PAE at the endpoint.

### Safety

The incidence of adverse events was low (*n* = 6), and all cases were classified as mild, causing only minor discomfort, without disrupting daily activities or requiring discontinuation of use. Five participants reported a tingling sensation following AZA application, while one participant reported pruritus. Participants who experienced facial dryness or sensitivity, such as ‘tingling’ and/or burning when using AZA, were advised to temporarily limit application to once daily and/or were reoriented on the amount of product to be applied to the face and side of the neck. When these side effects were controlled, these participants were advised to resume application twice daily.

## Discussion

In the present study, we observed a significant increase in IL-6 and IL-8 expressions within acne lesions compared with healthy skin, supporting the heightened inflammatory milieu associated with AFA. To our knowledge, evidence for immune pathways in AFA is extremely limited. Our results therefore provide novel insights into the local inflammatory mechanisms underlying this condition, particularly regarding the upregulation of IL-6 and IL-8 and their reduction following AZA treatment.

We previously investigated serum ILs in AFA but found no significant systemic elevation, reinforcing the idea that the inflammatory pathways involved in AFA are not reliably reflected in peripheral circulation. In that study, IL-6 was elevated only in women with obesity and with AFA, a finding likely attributable to the combined effects of obesity-related and systemic inflammation.^16^

Owing to the novelty of this study, no histological data for comparison were found in the literature. We also did not find any evaluations of IL expression in AFA skin, precluding a broader discussion. A Brazilian study employing immunohistochemistry revealed Toll-like receptor (TLR)-2 expression in sebocytes of the infundibulum in the pilosebaceous unit of patients with AFA.^17^ There was a predominance of TLR-2 in the periphery of the sebaceous glands in acne lesions and perilesional regions. In this study, AZA was effective in reducing TLR-2 expression, accompanied by clinical improvement in acne lesions.

A study evaluating IL-8 expression in inflammatory AV demonstrated significantly higher IL-8 immunoreactivity in lesional compared with nonlesional skin.^18^ The authors also reported pronounced IL-8 staining in dermal endothelial cells and within the neutrophilic inflammatory infiltrate, findings that correlated with increased dermal angiogenesis and the overall intensity of the inflammatory response.

*In vitro* data further highlight the central role of IL-8 in acne-­associated inflammation.^19^ This cell-based assay demonstrated that keratinocyte stimulation with bacterial components relevant to acne pathogenesis, such as lipoteichoic acid, peptidoglycan and lipopolysaccharide, induces robust IL-8 production through activation of TLR-2 and TLR-4 pathways. Inhibition of these receptors markedly reduced IL-8 secretion, and zinc gluconate suppressed both IL-8 release and the upregulation of TLR-2/TLR-4 expression. These findings reinforce IL-8 as a key mediator linking innate immune activation to the early inflammatory cascade in acne.

In patients with acne, keratinocytes, sebocytes, dendritic and inflammatory cells, monocytes and macrophages express TLR-2.^20,21^ This receptor is involved in comedogenesis and inflammation by activating nuclear transcriptional factors, such as nuclear factor kappa B (NF-κB), triggering the production of several cytokines, including IL-1α, IL-8 and IL-12, and activation of other cells, such as macrophages, natural killer cells and neutrophils.^22,23^ IL-8 acts as an important proinflammatory cytokine and a powerful chemoattractant.^24^ IL-6 secretion is stimulated by certain bacterial lipopolysaccharides, second messengers, cytokines and growth factors.^25^ This cytokine facilitates neutrophil trafficking and the production of other cytokines, proteases and free radicals. IL-6 can also induce T-cell differentiation, B-cell maturation and immunoglobulin production.^25^

In the present study, treatment with AZA significantly reduced the expression of both IL-6 and IL-8 in skin affected by inflammatory AFA lesions, suggesting a potential anti-inflammatory effect. AZA has been shown to modulate the inflammatory response in normal human keratinocytes by suppressing the mRNA expression of IL-1β and IL-6, as well as tumour necrosis factor-α, while concomitantly inducing peroxisome proliferator-activated receptor-γ mRNA expression and enhancing its transcriptional activity.^26^ This mechanism is associated with NF-κB inhibition, reducing the production of proinflammatory cytokines.^27^ In addition to these cytokine-related mechanisms, it is also possible that the improvements observed in our study reflect broader, nonspecific anti-inflammatory effects of AZA, rather than modulation of IL pathways alone.

Clinically, the reduction in inflammatory mediators observed in our study was accompanied by meaningful improvements in acne severity. Treatment with AZA resulted in progressive improvement after 6 months, with reductions in overall IGA scores, attenuation of erythema and better global clinical assessment. These findings were also evident in the photographic comparisons, in which patients showed fewer inflammatory lesions, improved AMH and a visible reduction in PAE at the study endpoint.

One strength of this study is its integrated design, combining clinical assessment with histological and immunohistochemical analyses. The use of paired pre- and post-treatment biopsies enabled direct evaluation of tissue-level changes, while ImageJ-based quantification reduced observer bias and increased measurement reliability. However, this study has some limitations, including a small sample size and limited population demographics, which may limit the power and generalizability of the findings; the limited number of observations may not adequately capture the full clinical variability of AFA. It is plausible that IL-6 and IL-8 levels fluctuate according to differences in lesion morphology, severity, hormonal background or other patient-specific factors. Our limited number of observations may therefore not fully capture the range of cytokine expression profiles present in the wider AFA population, which should be considered when interpreting these findings. Moreover, the study focused on IL-6 and IL-8 as inflammatory markers, but no further investigation was conducted to broaden the understanding of AFA pathophysiology. The study population lacked diversity, and factors such as hormonal variations, diet and lifestyle were not extensively analysed, which may have influenced the results.

In conclusion, this study further clarifies the dermatopathology of AFA, suggesting that mild-to-moderate cases may primarily involve localized peripheral skin inflammation. A significant reduction in IL-6 and IL-8 expression was observed after 6 months of treatment, correlating with clinical improvement. These results indicate that AZA may exert anti-inflammatory effects and offer therapeutic value in AFA. However, further research involving larger sample sizes, diverse populations and different treatment options is needed to expand on these results and explore alternative therapeutic approaches.

## References

[1] Goulden V, Stables GI, Cunliffe WJ. Prevalence of facial acne in adults. J Am Acad Dermatol 1999; 41:577–80. 10495379

[2] Poli F, Dreno B, Verschoore M. An epidemiological study of acne in female adults: results of a survey conducted in France. J Eur Acad Dermatol Venereol 2001; 15:541–5. 11843213 10.1046/j.1468-3083.2001.00357.x

[3] Bagatin E, Freitas THP, Rivitti-Machado MC et al Adult female acne: a guide to clinical practice. An Bras Dermatol 2019; 94:62–75. 30726466 10.1590/abd1806-4841.20198203 PMC6360964

[4] Branisteanu DE, Toader MP, Porumb EA et al Adult female acne: clinical and therapeutic particularities (review). Exp Ther Med 2022; 23:151. 35069832 10.3892/etm.2021.11074 PMC8753972

[5] Dagnelie MA, Poinas A, Dréno B. What is new in adult acne for the last 2 years: focus on acne pathophysiology and treatments. Int J Dermatol 2022; 61:1205–12. 35521784 10.1111/ijd.16220

[6] Jin Z, Song Y, He L. A review of skin immune processes in acne. Front Immunol 2023; 14:1324930. 38193084 10.3389/fimmu.2023.1324930 PMC10773853

[7] Akamatsu H, Komura J, Asada Y et al Inhibitory effect of azelaic acid on neutrophil functions: a possible cause for its efficacy in treating pathogenetically unrelated diseases. Arch Dermatol Res 1991; 283:162–6. 1867478 10.1007/BF00372056

[8] Vargas-Diez E, Hofmann MA, Bravo B et al Azelaic acid in the treatment of acne in adult females: case reports. Skin Pharmacol Physiol 2014; 27:18–25. 10.1159/000354889 24280645

[9] Nazzaro-Porro M, Passi S. Identification of tyrosinase inhibitors in cultures of Pityrosporum. J Invest Dermatol 1978; 71:205–8. 99481 10.1111/1523-1747.ep12547184

[10] Nazzaro-Porro M, Passi S, Picardo M et al Beneficial effect of 15% azelaic acid cream on acne vulgaris. Br J Dermatol 1983; 109:45–9. 6222755 10.1111/j.1365-2133.1983.tb03990.x

[11] Corrêa MP, Correia-Silva RD, Sasso GRS et al Expression pattern and immunoregulatory roles of galectin-1 and galectin-3 in atopic dermatitis and psoriasis. Inflammation 2022; 45:1133–45. 35031944 10.1007/s10753-021-01608-7

[12] US Food and Drug Administration . Acne Vulgaris: Establishing Effectiveness of Drugs Intended for Treatment. Guidance for Industry [Internet]. Silver Spring (MD): FDA, 2018. Available at: https://www.fda.gov/media/71152/download (last accessed 6 January 2025).

[13] Ma L, Huang X, Qiu Y et al Analysis of facial redness by comparing VISIA and YLGTD. Skin Res Technol 2023; 29:e13356. 37522504 10.1111/srt.13356 PMC10280608

[14] Chen Y, Hua W, Li A et al Analysis of facial redness by comparing VISIA® from Canfield and CSKIN® from Yanyun Technology. Skin Res Technol 2020; 26:696–701. 32196761 10.1111/srt.12856

[15] Kollias N, Stamatas GN. Optical non-invasive approaches to diagnosis of skin diseases. J Investig Dermatol Symp Proc 2002; 7:64–75. 10.1046/j.1523-1747.2002.19635.x 12518795

[16] Bomfim DP, da Rocha MAD, Sanudo A et al Adult female acne associated with normal levels of serum interleukins. Int J Dermatol 2024; 64:1277–8. 39482782 10.1111/ijd.17556

[17] Rocha MAD, Guadanhim LRS, Sanudo A et al Modulation of Toll-Like Receptor-2 on sebaceous gland by the treatment of adult female acne. Dermatoendocrinol 2017; 9:e1361570. 29484093 10.1080/19381980.2017.1361570 PMC5821154

[18] Abd El All HS, Shoukry NS, El Maged RA et al Immunohistochemical expression of interleukin 8 in skin biopsies from patients with inflammatory acne vulgaris. Diagn Pathol 2007; 2:4. 17263887 10.1186/1746-1596-2-4 PMC1797156

[19] Suvanprakorn P, Tongyen T, Prakhongcheep O et al Establishment of an anti-acne vulgaris evaluation method based on TLR2 and TLR4-mediated interleukin-8 production. In Vivo (Brooklyn) 2019; 33:1929–34. 10.21873/invivo.11687 PMC6899138 31662521

[20] Pivarcsi A, Koreck A, Bodai L et al Differentiation-regulated expression of Toll-like receptors 2 and 4 in HaCaT keratinocytes. Arch Dermatol Res 2004; 296:120–4. 15148609 10.1007/s00403-004-0475-2

[21] Fathy A, Mohamed RW, Ismael NA et al Expression of toll-like receptor 2 on peripheral blood monocytes of patients with inflammatory and noninflammatory acne vulgaris. Egypt J Immunol 2009; 16:127–34. 20726329

[22] Kim J . Review of the innate immune response in acne vulgaris: activation of toll-like receptor 2 in acne triggers inflammatory cytokine responses. Dermatology 2005; 211:193–8. 16205063 10.1159/000087011

[23] Selway JL, Kurczab T, Kealey T et al Toll-like receptor 2 activation and comedogenesis: implications for the pathogenesis of acne. BMC Dermatol 2013; 13:10. 24011352 10.1186/1471-5945-13-10 PMC3846817

[24] Sukkar M, Xie S, Khorasani N et al Toll-like receptor 2, 3, and 4 expression and function in human airway smooth muscle. J Allergy Clin Immunol 2006; 118:641–8. 16950283 10.1016/j.jaci.2006.05.013

[25] Ataie-Kachoie P, Pourgholami MH, Richardson DR et al Gene of the month: interleukin 6 (IL-6). J Clin Pathol 2014; 67:932–7. 25031389 10.1136/jclinpath-2014-202493

[26] Mastrofrancesco A, Ottaviani M, Aspite N et al Azelaic acid modulates the inflammatory response in normal human keratinocytes through PPARgamma activation. Exp Dermatol 2010; 19:813–20. 20545756 10.1111/j.1600-0625.2010.01107.x

[27] Briganti S, Flori E, Mastrofrancesco A et al Azelaic acid reduces the senescence-like phenotype in photo-irradiated human dermal fibroblasts: possible involvement of PPARγ. Exp Dermatol 2013; 22:41–7. 23278893 10.1111/exd.12066

